# Editorial: Long-term consequences of pediatric traumatic brain injury: Improved understanding to help young patients survive and thrive

**DOI:** 10.3389/fneur.2022.1011998

**Published:** 2022-09-09

**Authors:** Jimmy Huh, Bridgette D. Semple, Ramesh Raghupathi

**Affiliations:** ^1^Department of Anesthesiology and Critical Care, Children's Hospital of Philadelphia, Philadelphia, PA, United States; ^2^Perelman School of Medicine, University of Pennsylvania, Philadelphia, PA, United States; ^3^Department of Neuroscience, Central Clinical School, Faculty of Medicine, Nursing & Health Sciences, Monash University, Melbourne, VIC, Australia; ^4^Department of Neurobiology and Anatomy, College of Medicine, Drexel University, Philadelphia, PA, United States

**Keywords:** pediatric, traumatic brain injury, outcome, chronic, long-term, diffuse axonal injury (DAI), cognition, psychosocial

Improvements in pediatric neurocritical and neurosurgical care have improved overall survival rates for infants, children, and adolescents who suffer a traumatic brain injury (TBI). However, as these survivors age into adolescence and adulthood, many are afflicted with cognitive deficits and behavioral problems, such as social impairments, aggression and hyperactivity, and emotional disorders. Pre-injury characteristics, such as age, race/ethnicity, family, and environmental influences, can affect post-traumatic chronic sequelae. Recent studies have also shown that sex may influence long-term outcomes after TBI at different stages of maturation. This Research Topic, entitled “*Long-term consequences of pediatric traumatic brain injury*” provides excellent reviews, preclinical and clinical studies on the effects of pediatric TBI at different stages of development on chronic histopathologic, cognitive, and psychosocial outcomes.

A comprehensive review by Serpa et al. on the “*Pathophysiology of Pediatric Traumatic Brain Injury*” summarizes the available pre-clinical studies demonstrating age-at-injury differences in the acute pathophysiology of calcium accumulation, glucose metabolism, cerebral blood flow, mitochondria, and inflammatory responses in the injured brain, which may potentially drive differences in long-term outcomes. The authors also review pre-clinical models of mild, repetitive mild, and more severe TBI during cerebral maturation, and the effects on chronic outcome trajectories. An interesting discussion by this group focuses on the role of exercise on the response to pediatric TBI. Children and adolescents are at a high risk for mild TBI, with adolescents also being exposed to repetitive mild brain injuries (i.e. sports-related concussions). A plethora of studies have demonstrated the negative long-term consequences of sports-related mild and repetitive mild TBI and the authors highlight the role of exercise both before and after the TBI in modulating outcomes. Exercise in adolescents has demonstrated beneficial cognitive, psychosocial and immunological responses which may potentially affect responses to brain injury. The authors highlight important studies where adolescent rodents exposed to pre-injury exercise demonstrated protection from cognitive and motor deficits associated with attenuated neuroinflammatory and apoptotic responses compared to sedentary rodents following TBI. While these preclinical studies were performed in males, further studies need to be addressed in females as the response may be different. Ferguson et al. (“*Sex Differences in Neurophysiological Changes Following Voluntary Exercise in Adolescent Rats*”) studied the effects of exercise and sex and reported that female adolescent rats ran farther and for longer periods of time than male adolescent rats, associated with an acute increase in brain-derived neurotrophic factor (BDNF) in only the exercised females. These data suggest the importance of studying exercise-dependent changes on the adolescent brain in both males and females to better understand the long-term recovery response of adolescents following TBI. From a clinical perspective, a relationship between activity and age was examined by Iverson et al. who conducted a literature review on clinical studies on male adolescents, titled “*Age of First Exposure to Contact and Collision Sports and Later in Life Brain Health: A Narrative Review*.” They found that involvement in contact sports before the age of 12 years was not associated with worse chronic cognitive functioning or mental health problems in current high school or college athletes or in middle-aged men who played high school football. These authors concluded that results from studies on former NFL players are mixed, and do not currently support the theory that exposure to tackle football before the age of 12 is associated with long-term cognitive or mental health impairment. While clearly no one advocates for any brain injury during contact sports, further studies on long-term outcome are evidently needed. Importantly, chronic outcome data on female athletes remain sorely lacking.

Pre-injury circumstances such as age, family and environmental influences may also impact long-term outcomes following TBI. In an article by Doust et al. titled “*Age-at-Injury Determines the Extent of Long-Term Neuropathology and Microgliosis After a Diffuse Brain Injury in Male Rats*,” diffuse TBI at juvenile, adolescent, young adult, or mature adult ages (17 days - 6 months old) in male rats survived until 10 months, when chronic histopathology was analyzed. Regardless of the age when TBI occurred, increased neuropathologic changes and microglial activation was observed, while increased astrocyte activation was not seen at 10 months of age in the injured animals. The extent of dendritic neurofilament pathology and proportion of microglial colocalization with functional markers of phagocytosis (CD68) and alternative activation (TREM2) after diffuse TBI demonstrated an age-at-injury effect in a region-specific manner. Clearly, additional studies on the effect of age and time since injury as well as sex are needed.

It is well known that infants and young children exposed to early life stress alone are at risk for developing long-term cognitive and psychosocial impairments at adulthood. An engaging review by Parker et al. titled “*Traumatic Injury to the Developing Brain: Emerging Relationship to Early Life Stress*,” considers the preclinical literature on the effects of early life stress and subsequent acquired brain injury (TBI, stroke and hypoxia-ischemia) during early brain development. Data demonstrate that early life stress “primes” the immune cells of the brain and periphery to elicit a heightened inflammatory response following subsequent brain injury, that may affect chronic outcomes. For example, in a rodent model of neonatal neglect, using a maternal separation model as a form of early life stress, a subsequent TBI at adolescence or adulthood was associated with worsening cognitive outcome, increased microglial activity and increased pro-inflammatory IL-1β cytokine levels (1–3). Elsewhere in this Research Topic, in a clinical study by Ewing-Cobbs et al. titled “*As Time Goes by: Understanding Child and Family Factors Shaping Behavioral Outcomes After Traumatic Brain Injury*,” a child with greater pre-injury executive dysfunction, or one who lives in a family with lower income, who sustained a TBI had a higher risk for both emotional symptoms and conduct problems at 12 months post-injury. Furthermore, female sex, and worse family dysfunction were associated with worse emotional symptoms; while younger age and pre-existing emotional/behavioral problems were associated with worse conduct problems at 12 months post-injury. At long-term follow-up between 12 and 36 months post-injury, emotional symptoms worsened, while conduct problems stabilized. Interesting, TBI severity had no effect on these aspects of chronic psychological outcome. In a different study by Jones et al. titled “*Parent and Teacher-Reported Child Outcomes Seven Years After Mild Traumatic Brain Injury: A Nested Case Control Study*,” children who sustained mild TBI were followed up to 7 years post-injury. The authors report significantly greater emotional symptoms, conduct problems, hyperactivity/inattention, and executive dysfunction observed by parents compared to non-TBI controls. Social deficits are becoming increasing recognized in young survivors of TBI as they progress to adolescence and adulthood. Semple and Raghupathi provide a timely review titled “*A Pro-social Pill? The Potential of Pharmacological Treatments to Improve Social Outcomes After Pediatric Traumatic Brain Injury*”. For example, one of the pre-clinical studies they discuss is the effective treatment of the neuropeptide oxytocin administered intranasally 4-5 weeks post-injury to ameliorate social recognition deficits in the adolescent stage following diffuse TBI in the neonate rat (4).

A clinical study on moderate and severe pediatric TBI by Kennedy et al. titled “*Moderate and severe TBI in children and adolescents: The effects of age, sex, and injury severity on patient outcome 6 months after injury*” demonstrated that Glasgow Coma Scale (GCS) was the most powerful predictor of outcome. Infants had the highest mortality rate and trended toward the worst outcome where abusive head trauma was common, while sex had no effect. Secondary injuries of hypoxia, hypotension, and hypothermia was associated with worse GCS and higher mortality. Finally, the Research Topic includes 2 clinical studies on long-term outcomes associated with pediatric diffuse axonal injury (DAI). In a study by Wilde et al. titled “*A Preliminary DTI Tractography Study of Developmental Neuroplasticity 5–15 Years After Early Childhood Traumatic Brain Injury*,” early diffuse TBI was associated with hypertrophic cingulum bundles, and the number of tract streamlines was inversely correlated with the age-at-injury. However, the streamline density had no effect on executive function during the chronic post-traumatic period. In contrast, while the streamline density of the perforant pathway had no correlation to age-at-injury effects, streamline density of the left perforant pathway was positively correlated with verbal memory scores during the chronic post-traumatic period. In another study by Lang et al. titled “*Trajectory of Long-Term Outcome in Severe Pediatric Diffuse Axonal Injury: An Exploratory Study*” where children with DAI were followed up to 10 years post-injury, early fever and extensive DAI on MRI were associated with worse long-term outcomes. However, of the surviving children who had follow-up for 10 years after injury, many children made a favorable recovery. Collectively, these studies suggest that there is potential for some beneficial neuroplasticity in survivors following diffuse pediatric TBI.

In conclusion, this Research Topic highlights the importance of long-term outcome following early pediatric TBI- while most survive, many are left with long-term cognitive, psychologic, and social problems. The next step is the obvious: continue on-going translational studies to better understand the mechanisms and factors associated with poor chronic functional outcomes to ultimately discover therapeutic strategies to promote beneficial chronic functional outcomes so these children can “survive and thrive”!

## Author contributions

JH, BS, and RR were all involved in substantial contributions to the conception or design of the work, acquisition, analysis, or interpretation of data for the work, drafting the work or revising it critically for important intellectual content, provided approval for publication of the content, and agree to be accountable for all aspects of the work in ensuring that questions related to the accuracy or integrity of any part of the work are appropriately investigated and resolved. All authors contributed to the article and approved the submitted version.

## Conflict of interest

The authors declare that the research was conducted in the absence of any commercial or financial relationships that could be construed as a potential conflict of interest.

## Publisher's note

All claims expressed in this article are solely those of the authors and do not necessarily represent those of their affiliated organizations, or those of the publisher, the editors and the reviewers. Any product that may be evaluated in this article, or claim that may be made by its manufacturer, is not guaranteed or endorsed by the publisher.
